# SEND: a suite of tools for the easy sharing of linked biological data

**DOI:** 10.1093/database/baaf068

**Published:** 2025-10-29

**Authors:** Rodolfo S Allendes Osorio, Yi-An Chen, Kenji Mizuguchi

**Affiliations:** Premium Research Institute for Human Metaverse Medicine (WPI-PRIMe), The University of Osaka, 2-2 Yamadaoka, Suita-shi, Osaka 565-0871, Japan; AI Center for Health and Biomedical Research (ArCHER), National Institutes of Biomedical Innovation, Health and Nutrition, 3-17 Senrioka-Shinmachi, Settsu-shi, Osaka, 566-0002, Japan; AI Center for Health and Biomedical Research (ArCHER), National Institutes of Biomedical Innovation, Health and Nutrition, 3-17 Senrioka-Shinmachi, Settsu-shi, Osaka, 566-0002, Japan; AI Center for Health and Biomedical Research (ArCHER), National Institutes of Biomedical Innovation, Health and Nutrition, 3-17 Senrioka-Shinmachi, Settsu-shi, Osaka, 566-0002, Japan; Institute for Protein Research, The University of Osaka, 3-2 Yamadaoka, Suita-shi, Osaka 565-0871, Japan

## Abstract

Here, we introduce SEND (Share Easily bioiNformatics Data), a suite of tools that allow users to share heterogeneous and linked biological data, in an easy and highly customizable way. The code for SEND is freely available on GitHub (https://github.com/targetmine). Also, docker images of all different components of the system are directly available for download from DockerHub (https://hub.docker.com/search?q=rallendes; contact: Rodolfo S. Allendes Osorio, rodolfo.allendes.prime@osaka-u.ac.jp; Kenji Mizuguchi, kenji@protein.osaka-u.ac.jp). Links to additional figures/data available on a web site, or references to online-only supplementary data available at the journal’s web site.

## Introduction

The development of science is generally fostered by the sharing of new methods and techniques. However, when looking in particular at biological sciences, also required is to share the data as the results of research appropriately. This issue has been placed in the spotlight, particularly by the discussion and adoption of the FAIR principles in biological research [1], by which data need to be ‘Findable, Accessible, Interoperable, and Reusable’.

In the case of single, even sometimes considerably large, experiments, the use of public repositories is a very effective approach. Proof of this is the construction and maintenance of well-established databases such as the worldwide Protein Data Bank [2], GeneBank [3], and UniProt [4]. The use of such repositories also falls in line with the benefits involved in data sharing and the adherence to the FAIR principles, as described in [5].

Wilson et al. [5] provided details on how, when, and where different types of biological data should be shared; they presented concrete examples for general tabular data, genomics, proteomics, microscopy, and structural biology datasets.

However, recent trends in biological research usually consider the case in which multiple types of data need to be combined for analysis. Here, if we are to follow the FAIR principles and the suggestions given at [5], whenever possible we need to consider the use of generalistic platforms that could manage several different types of data, and such a platform should provide not only the means for users to share the data but also the tools that can ease their integration and access.

We believe InterMine [6] to be an example of this trend. Intermine is an open-source data warehouse platform used by several different groups to provide integrated access to data for multiple organisms and life science research areas. One implementation of this platform, TargetMine [7], an effort to support the drug discovery process, integrates data on genes, proteins, compounds, diseases, and so on, obtained from over 30 public databases and provides the ability to search for relationships between each item efficiently.

Even when platforms such as TargetMine [7] can be considered to adhere to the FAIR principles, some of its characteristics still prevent this approach from being more widely adopted by the biological science community. In particular, the development and deployment of such systems require computer science domain-specific knowledge, which often becomes a hurdle hard to overcome with limited time and budgets. Similar difficulties can be found when using generic database management systems, such as phpMyAdmin^1^ and MySQL Workbench,^2^ as these are specifically targeted to administrators, and usually assume some degree of knowledge on database systems.

As an alternative, we propose the implementation of SEND (Sharing of linkEd bioiNformatics Data), an intermediate solution between public repositories and customly developed ad hoc storage applications. Through the implementation of several interconnected components, SEND allows the integration, storage, retrieval, and online sharing of various types of custom datasets. With SEND, we aim to build a platform for ensuring the reusability of research data and the automatic generation of training data for machine-learning applications in the life science field.

We believe that SEND has the potential to support the sharing of a variety of biologically relevant information, including single omics, multiomics and highly heterogeneous datasets. Table 1 provides a brief summary of the composition of datasets that could potentially be shared through SEND.

**Table 1.** Sample types of datasets that could be shared using SEND

Type of dataset	Domain	Sample entities	Sample relations
Single omics	Transcriptome	Cell Gene	Cell to gene
	Proteome	Protein	Protein protein interaction
Multiomics	Transcriptome + metabolome	Gene Metabolite	Metabolic pathway
Heterogeneous	Transcriptome + disease	Cell Gene Disease	Cell to gene Gene to disease

For example, for the case of single omics datasets, entities such as *Genes* and *Proteins* can be shared [including the relations that could exist between instances of the same entity, such as protein–protein interaction (PPI) networks]. At the bottom end, heterogeneous datasets that include, e.g. both genomic and disease-related information, can also be shared using SEND.

## SEND

### Motivation

Let us consider the case of studying the production mechanisms of the amyloid precursor protein (APP) by combining different sources of information, in a way similar to that introduced in [8]. We will consider information to be available in the form of two files that contain binary PPIs, rather than pathways as in the original study, and a series of siRNA screens. Even when both the interaction network and the siRNA screens could individually provide important insights into the research question, greater insights can be achieved by defining different ways in which these can be combined.

For example, we can use the siRNA screen values to add attributes (e.g. in the form of weights) to the nodes (i.e. proteins) of the PPI network. By defining different ways in which these weights are generated, we can effectively generate a series of different, parametrized versions of the original PPI network.

In this case, it is important for data generators not only to be able to share the original data but also to include ways in which they can also share the different links between them. This is to enable the sharing of both the model that describes the relation between different entities and the instances (data) that populate the model.

At the opposite end, data consumers search for a completely different set of functionality. They are presented with an already constructed model, populated with data; thus, their main concern usually relates to being able to effectively assess how different sections of the model/data could be relevant for their own work; once identified, they need the tools to extract it from the complete dataset.

In the context of our previous example, the data generator is interested in defining the properties of the elements (Proteins), the relations between them, and the multiple weights that could be associated with each edge, together with actually providing a list of proteins, relations, and weights that comply with this model. The data enquirer is interested, e.g. in extracting only the proteins in the model, or a single weight from the list of available ones.

In our opinion, it is clearly important to allow data generators the tools that allow them to store the data together with the relations that they define among them. At the same time, we need to consider the needs of a data querier, who needs to be provided with integrated access to it, together with the tools required to extract all or different subsets of the data. This means the definition of an integrated framework, which can handle two different types of users, on a shared data instance.

### SEND’s definition

As a solution, here we introduce SEND, an integrated platform that:

- supports data providers in the definition of custom biological models, including elements and interactions; and with the storage of the corresponding data seamlessly into a database;
- supports the easy sharing of the customly created models and data
- supports the access to the stored models and data to data users, also referred here as data enquirers.

**Figure 1.**
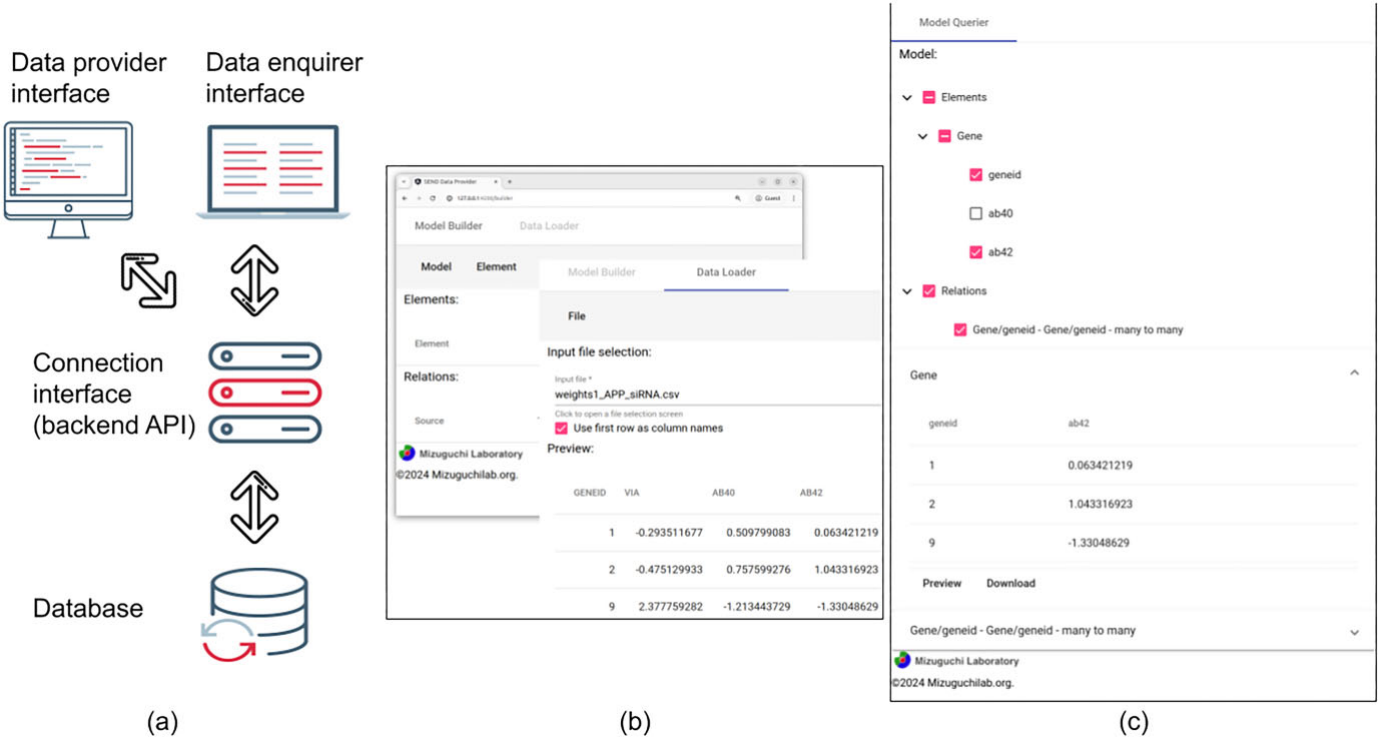
(a) Overall structure of SEND, showing the interactions between the different components of the framework. (b) Sample interface of the Data Provider component. (c) Sample interface of the Data Enquirer component.

Figure 1a shows the overall architecture of SEND, and how its different components are linked together in order to implement our solution to the problem of data storage and sharing.

At the bottom of the diagram shown in Fig. 1a, the first component of the SEND platform is a database that acts as a joint repository for all the data to be shared. In our example, both PPI and siRNA data are stored together in a single, integrated database. Notice that the database does not only store the individual interactions and screens but also the relations between these two different data elements.

On the top of Fig. 1a, the *Data provider interface* includes the functionality used for the definition of elements and relations to be stored, together with the actual upload of the data. In our example use case, information can be stored through the definition of a single *Gene* element. Here, an ‘element’ represents any biological object, and its definition requires a unique ID and any sets of attributes (in our example, the siRNA readouts associated with each gene). Additionally, PPI information is integrated by defining a single relation between gene elements, where a ‘relation’ can be defined by linking together the unique IDs of (different) elements. After the model has been finished, the actual values are then loaded into the database from a CSV.

Since all of the tools required to define the underlying biological model to be stored are provided through a graphical user interface (GUI), data providers do not need to use sometimes complicated SQL syntax (or other types of programming code) in order to define or populate the database. At the same time, simply changing the details of the model allows SEND to satisfy the needs of many different users, bringing together both the advantage of using a single tool with the potential of diverging into several different instances of ad hoc storing solutions.

Similar to the data provider, the Data Enquirer uses a different interface (Fig. 1b) to select, display, and export different views of the data. The enquirer interface loads the previously generated biological model and populates it with the data also stored in the shared database.

Connecting the user interfaces with the data and relation storage, our platform includes a single *Connection interface* (also referred to as backend API, Fig. 1). This interface allows the data provider to store the integrated biological model and upload the corresponding data.

### Implementation

A series of different technologies are used for the implementation of the different components of SEND. In the following, we provide some technical implementation details for the platform.

We use PostgreSQL as the integrated repository (*Database* element) for all data handled by the platform, as this allows us to take advantage of all the benefits of a well-established manager in terms of performance and data integrity (atomicity, consistency, isolation, and durability or ACID compliance). As a general rule, each instance of data sharing begins with an empty database for Data Providers. However, only databases that have already been populated can be used in conjunction with the Data Enquirer interface.

Both the Data Provider and Data Enquirer interfaces are implemented in web applications using the Angular^3^ framework for web development. As such, they can be accessed via any major web browser. Using navigation elements commonly used whilst browsing the internet, we believe users will quickly adapt to the controls used by SEND to define biological models and upload associated data.

Finally, the *Connection Interface* (also referred to as API within this document) is implemented using the Express^4^ framework. However, unlike the provider and enquirer components, the connection interface is meant to be a bridge between the different elements of SEND. It is not meant to be used directly by a user. The connection interface implements a REST API that, on one end, replies to the HTTP requests generated by the provider and enquirer web applications and, on the other, connects and performs operations directly to the database.

Despite the number of different software components involved in the development of SEND, the implementation details and the way in which they are interconnected are completely transparent to the different end users. Just as an example, although PostgreSQL is used as the underlying database management system of SEND, users do not need to download/install or have any direct interaction with that particular tool; all the interaction is triggered by the graphical interface, and handled under the hood by SEND.

A detailed explanation of the use of the different functionalities exposed by SEND is provided in the supplementary user guide.

Since the data and interactions shared by each Data provider are unique, each populated instance of SEND (each database and Data Enquirer interface) will be automatically customized. In order to generate and share these customized versions, we take advantage of the possibilities offered by Docker^5^ for the sharing of software environments as images.

SEND is distributed as a series of Docker images; in order to use it, data providers download the images required for the generation of a new shared model (data provider, connection interface, and database) and, in turn, generate a new, custom Docker image of the data enquirer interface. In turn, Data enquirers download the custom-created data enquirer interface and database, together with the common connection interface. In both situations, the only prerequisite for users to use SEND is to have a working installation of Docker.

In addition to the Docker images, the source code for all of SEND’s components is also freely available under an MIT Licence on GitHub.^6^ We expect users who require an even higher level of customization could use SEND’s components as a starting point for their own sharing solutions.

## Conclusions

Here, we have introduced SEND, a highly customizable platform for integrating, storing, and online sharing of biological data.

Through the interaction achieved by SEND’s four individual components, we provide a tailored solution for two different types of users: On the one hand, researchers that are looking for ways to share their heterogeneous datasets, and on the opposite end, users that wish to query these datasets subsequently.

The modular approach used for the implementation of SEND also provides users with a highly customizable environment for the sharing of data. Different types of data can be potentially combined into a single model. Also, multiple instances of identical types of data can be shared as multiple versions of a single model. Overall, SEND has the potential to adapt to the multiple needs of different researchers.

By leveraging the use of current technologies on software encapsulation, such as Docker images, together with the definition of interfaces that use known web interaction methods to mask more complex database interactions, we ensure that the level of proficiency in computer program development poses no hurdle to potential users of SEND. Table 2 summarizes some of the key aspects that we believe make SEND a valuable new addition for researchers looking to share their biological data. For example, the need for no programming and the handling of all operations through a GUI, the ease of installation, or even the overall knowledge required for the user to handle custom biological models are key strengths of SEND.

**Table 2.** Key aspects associated with SEND and how they compare with other traditional data sharing solutions currently available

	SEND	InterMine	phpMyAdmin/MySQL
Required knowledge	Data structure	Data structure, programming, server management	Data structure, DBMS, SQL syntax
Query	GUI	GUI	SQL query
Dealing with many-to-many relations	Auto	Auto	Manual
Programming	Unnecessary	Java	Unnecessary
Installation	Docker	PostgreSQL, Tomcat, Java	MySQL (MariaDB), PHP, Apache (httpd)
Data loading	GUI	Gradle, usually needs coding	GUI
Data sharing	Model file and database dump	Not designed for sharing	A dump of the database

Although in theory, there is no limit to the size of the dataset that SEND could manage (the underlying postgres data management is only limited by the actual size of the hard-drive used), we envision SEND as a solution for the sharing of medium-sized datasets, which we consider to be not more than a few gigabytes in total.

Rather than the overall size, we believe that the strength of SEND is its capability for the simple development of multiple biological models, which allows seamless integration of several small datasets into a single, integrated space, and overcomes the rigidity of existing systems that target larger datasets.

We believe that SEND has the potential to contribute to the overall problem of data sharing, in line with the FAIR principles, especially when considering highly interrelated data for which no current general-purpose store website is yet available.

## Supplementary Material

baaf068_Supplemental_File

## Footnotes

1
https://www.phpmyadmin.net/- Last accessed: 29 January 2024.

2
https://www.mysql.com/products/workbench/- Last accessed: 29 January 2024.

3
https://angular.io/- Last accessed: 22 December 2023.

4
https://expressjs.com/- Last accessed: 22 December 2023.

5
https://www.docker.com/- Last accessed: 22 December 2023.

6
https://github.com/- Last accessed: 22 December 2023.
