# Supplementary material for: SEND: a suite of tools for the easy sharing of linked biological data
Source: Database (Oxford). 2025 Oct 29;2025:baaf068. doi: 10.1093/database/baaf068 (PMC12569301; doi:10.1093/database/baaf068)
Supplement: baaf068_Supplemental_File [file baaf068_supplemental_file.pdf]

SEND Platform User Guide

This user guide provides introductory examples for using the *Data Provider* and *Data Enquirer* interfaces of the SEND data sharing suite.

1. System Requirements

Please notice that the different components of SEND have, in principle, the same system requirements when using docker images. Namely these requirements are:

- An available version of docker, with docker compose > 2.0.
- A modern web browser

The requirements to run each component from source are listed in the corresponding sub-sections of this document.

2. Data Provider

Overview

The *Data Provider* interface is the element of SEND available for users who want to share their data with others. The interface is composed of three different components (each implemented as an independent software element), namely the PostgreSQL `Database` component, the `Backend` component, and the `DataProvider` component.

A typical workflow for the *Data Provider* interface comprises the following steps:

1. Software installation (download)
2. Definition of a model
3. Model export
4. Upload of model data
5. Persistence of database
6. Model and Data sharing

In the following, we provide users with details on each of these steps, and provide sample models and data that can be used for testing. We believe these steps to match the highest number of potential users of our platform. A video that replicates these steps is freely available at https://vimeo.com/1112925039

Installation

The *Data Provider* interface is available and ready-to-use through docker images[1]:

1. The download and execution of all the components that comprise the *Data Provider* interface can be done via a single configuration file: the `docker-compose.yml` file for the component.

   The file (notice that you need the raw version) is freely available at the following link: `https://github.com/targetmine/send_DataProvider/blob/main/docker-compose.yml`. Simply click on the `download` button available to get a copy of the file.

   Alternatively, the file can also be downloaded by using the following terminal command -notice the link is slightly different to the one above, as we need the *raw* file and not the html wrapped version-:

```
:~/$ mkdir sendProvider
:~/$ cd sendProvider
:~/sendProvider$ wget
https://raw.githubusercontent.com/targetmine/send_DataProvider/refs/heads/main/docker-compose.yml
```

2. Create a sub-folder named `backup`. The model definition and database backup will be saved to this default directory by the *Data Provider* interface.

```
:~/sendProvider$ mkdir backup
```

3. Execute the command: `docker compose up -d`.
   The `docker-compose` file contains the instructions to (download and) execute the three containers that form the backbone of the *Data Provider* interface, namely the `Database`, `Backend` and `DataProvider` components. Also at this step a docker volume will be created. The volume will later be used to persist the database of the *Data Provider*.

```
:~/sendProvider$ docker compose up -d
[+] Running 5/5
 ✔ Network testeand_send_net  Created   0.1s
 ✔ Volume "send_volume"       Created   0.0s
 ✔ Container send_db          Started   0.5s
 ✔ Container send_backend     Started   0.8s
 ✔ Container send_builder     Started   0.7s
:~/sendProvider$
```

4. After the three containers for the application have been started, the application should be available through a web browser on address http://127.0.0.1:4200/

[1] An installation of docker is required to use this method. A guide to install docker in different platforms is available from the following website: https://docs.docker.com/get-docker/

Source code for the *Data Provider* component is publicly available on GitHub at https://github.com/targetmine/send_DataProvider. The *Data Provider* component of SEND is implemented using the Angular framework. To compile and execute the source code, a working installation of *npm* and *Angular* are required.

Definition of a Model

On load, the application should display an empty model, like the one from Fig. 2-1. In the following, we will give details on how to add new `Elements` and `Relations` to this empty model.

As an example, the final version of the model created through this section is available for download from file `backup/model.json` on the GitHub repository for `send_DataProvider`. Notice that the model can be directly loaded from this file by using option *Model -> Load Model.*

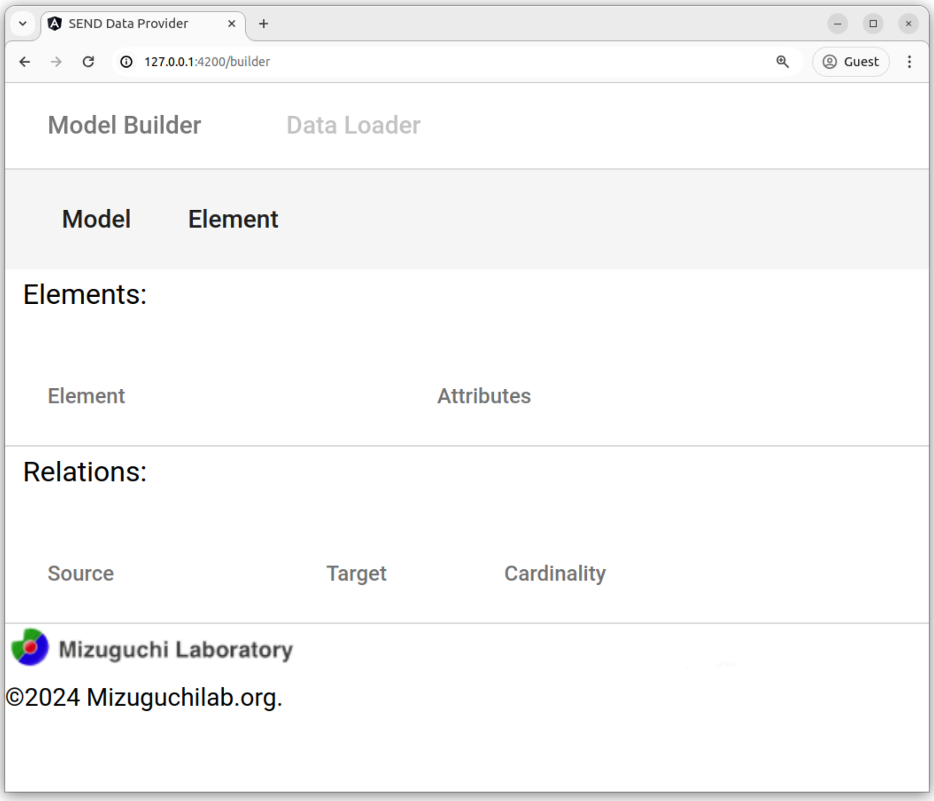

Figure 2-1. Data Provider interface with an initial empty model.

Elements

New *Elements* can be added to the empty model by clicking on the `Element` button followed by `Add Element`, completing the details required, and clicking on `Submit` on the pop-up window. This sequence of steps is shown in Fig. 2-2.

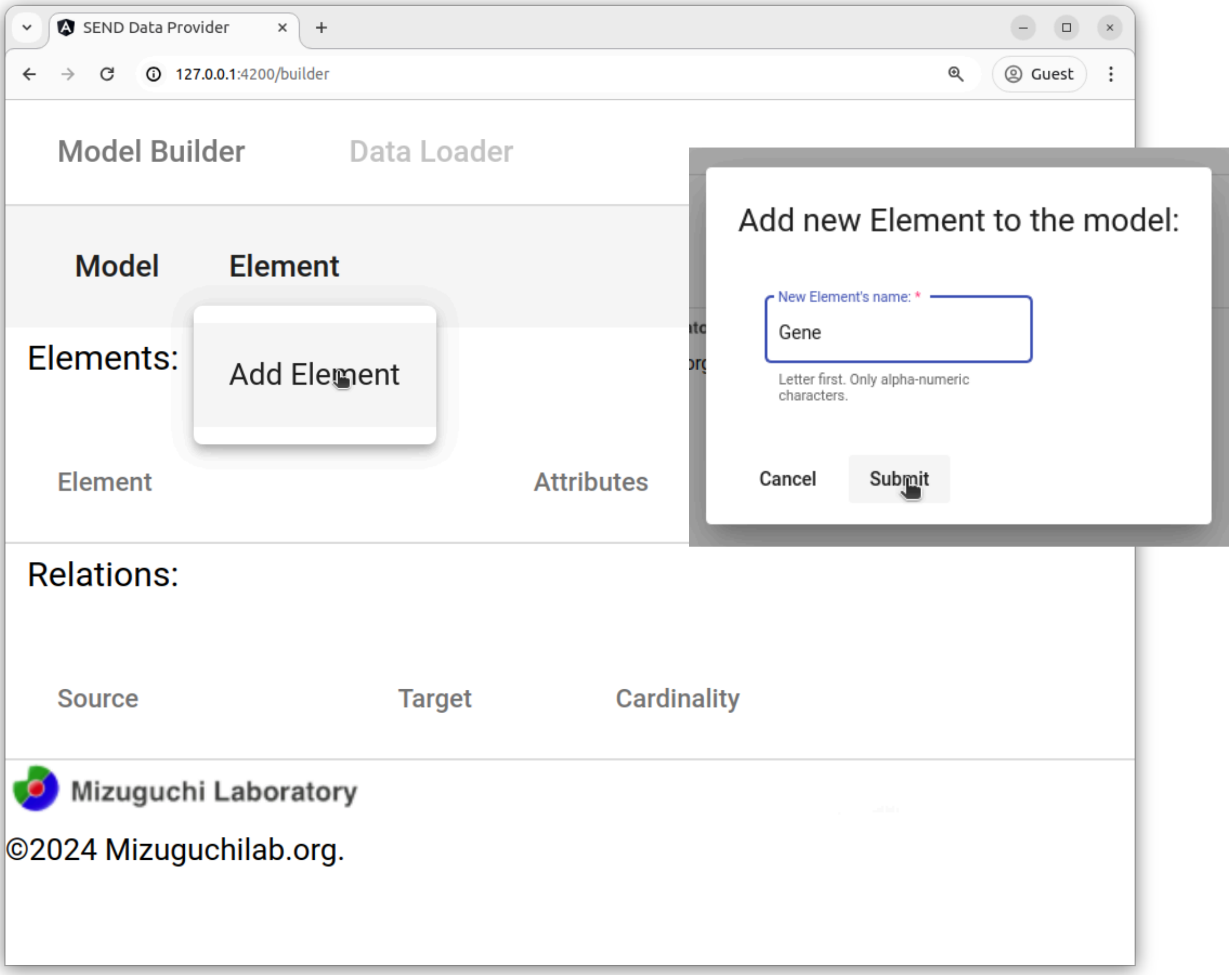

Figure 2-2. Sequence of steps required to add a new Element to the model. In the example, an element called "Gene" is added.

Element attributes

Multiple *Attributes* can be added to previously defined *Elements*. To add a new attribute, click the `Add` button located next to the element's name and follow the instructions presented in the pop-up window. Attributes that should have unique values can be indicated by selecting the corresponding checkbox (as shown in Fig. 2-3b).

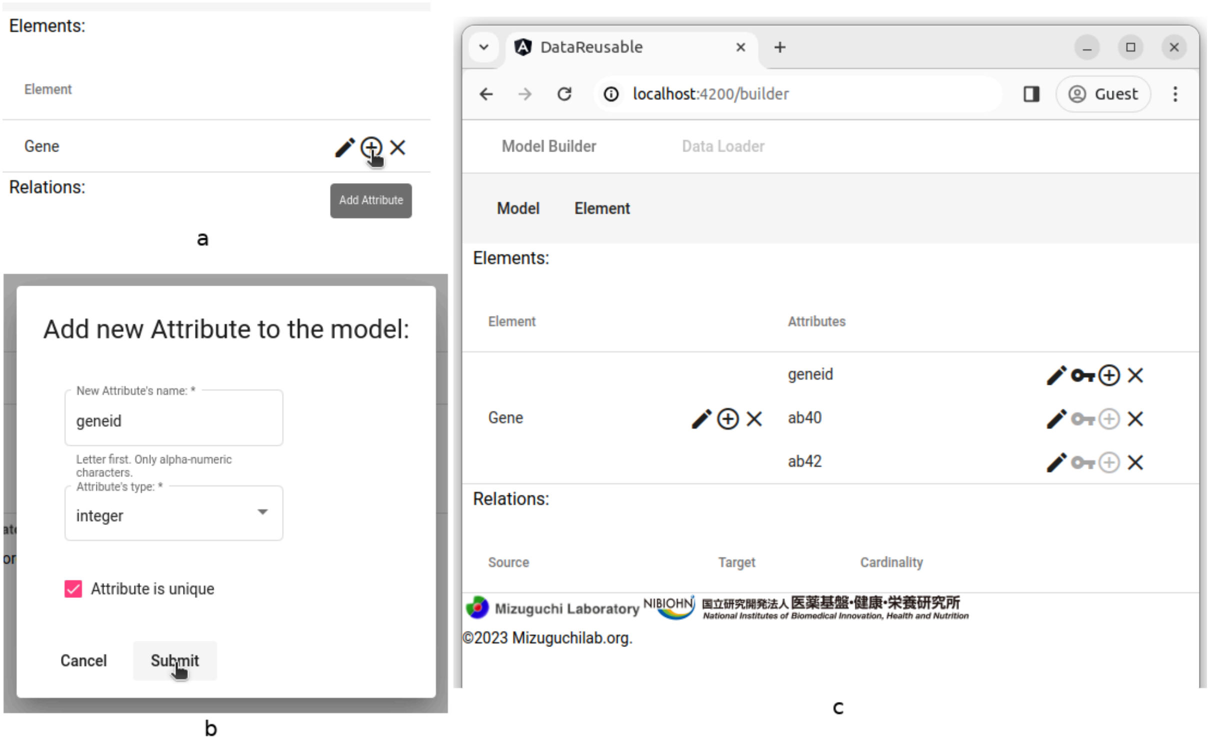

Figure 2-3. Adding attributes to previously created elements. a) Click the add button to add a new attribute. b) Indicate the name, type and whether the values for the new attribute need to be unique. c) Multiple attributes can be added to a single element.

Relations

In addition to *Elements* a model can also include a series of *Relations* between elements. Relations can be defined between the uniquely valued (key) attributes of two distinct elements. Relations can also be used to link an Element to itself (for example, when creating a model that includes data regarding a Protein Protein interaction network - PPI).

To add a new *Relation,* click on the `Add` button located next to the attribute that will be used as source for the relation. Then simply follow the instructions on the pop-up window to include the target's element and attribute and the relation's cardinality.

The *cardinality* property indicates how individual source and target elements are allowed to be related. The available values for cardinality are:

- one to one - each source can be related to a single target,
- one to many - one source can have many related targets, but each target is related to a single source, and
- many to many - a source can be related to multiple targets and each target can also be related to many sources.

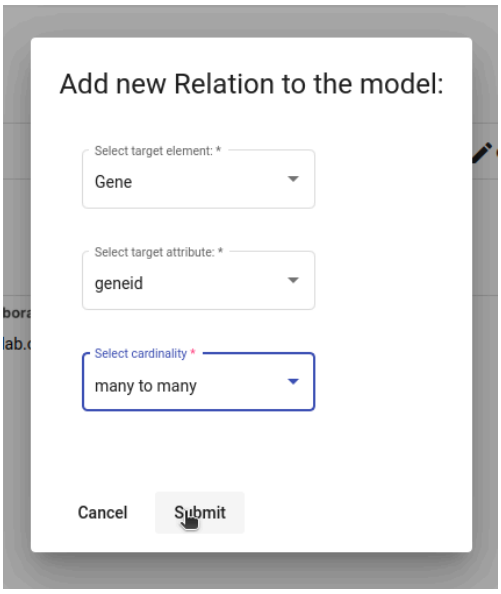

Figure 2-4. Pop-up window used to add Relations to the model.

Model Persistance

In order for the model to be shared with users of the *Data Enquirer* interface, users need to export the defined model before continuing with data uploading.

To export a model, users need to select the `Export model to file` option from the `Model` menu. Notice that the saved model does not include any data. For consistency, the model needs to be saved to a file called `model.json`.

Models can also be exported at any point during the normal execution of the *Data Provider* interface definition of a model; moreover, models can also be loaded into an empty session to continue their definition. A previously saved model can be loaded to the application by selecting the `Load Model` option from the same Menu.

Finish Model

After completing the details of the model, the structure needs to be saved to the underlying database before any actual data can be uploaded.

Click the `Model` button and select the `Finish Model and Upload Data` option. Click the `OK` button to confirm finishing the model building. You will be brought to the `Data Loader` tab for data importing.

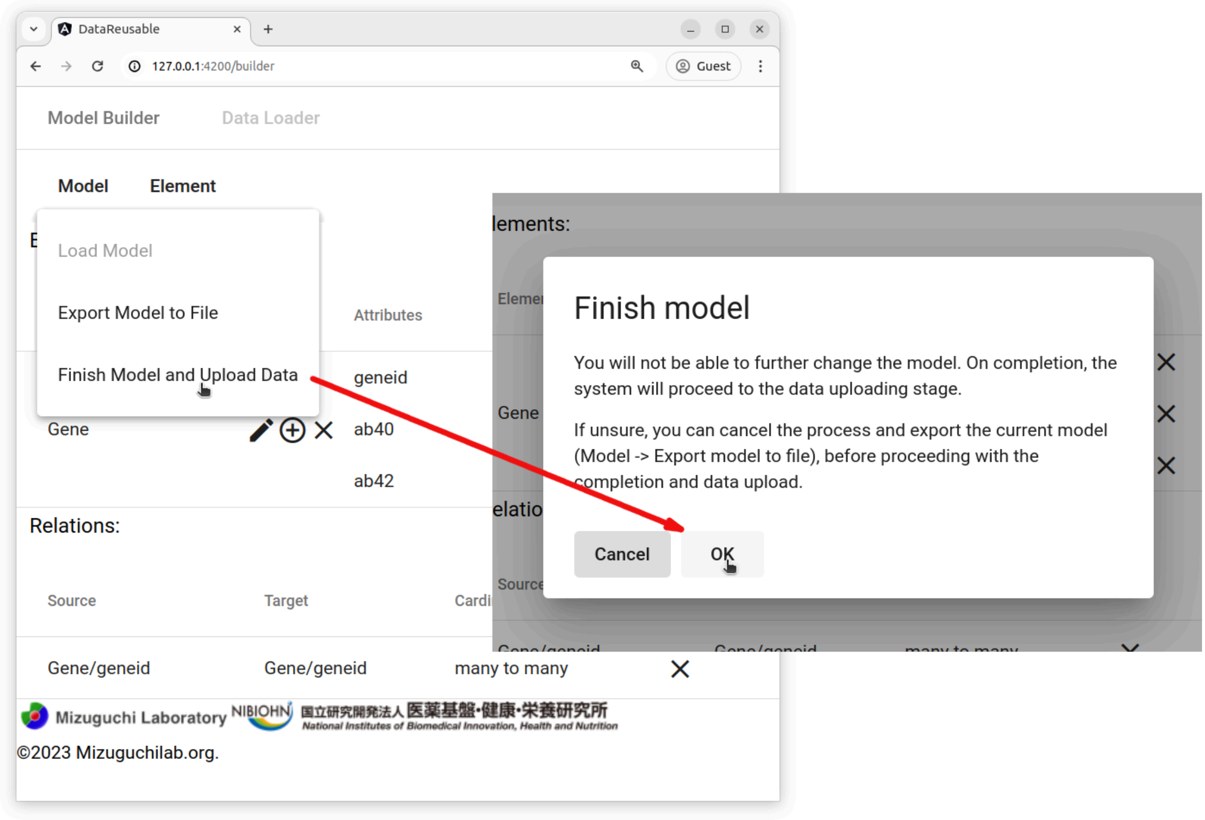

Figure 2-5. Finishing the definition of a Model before proceeding to upload the corresponding data.

Data upload

Data for the different elements and relations on the model is uploaded through the `Data Loader` tab. Once the Model has been defined, you can upload your data.

Select an input file from your PC[2], and you can see a preview of the contents below. Then, match the columns in the input file with the different attributes of a model's element and click the submit button to upload the data to the underlying database.

Two sample files with test data that can be used to populate the different elements and relations of the current model are available at `backup/elements.csv` and `backup/relations.csv` of the `send_DataBuilder` repository in GitHub.

The permanent links to download these files are:

```
https://github.com/targetmine/send_DataProvider/raw/refs/heads/main/back
up/elements.csv

https://github.com/targetmine/send_DataProvider/raw/refs/heads/main/back
up/relations.csv
```

[2] At this moment, data can be loaded only from CSV files.

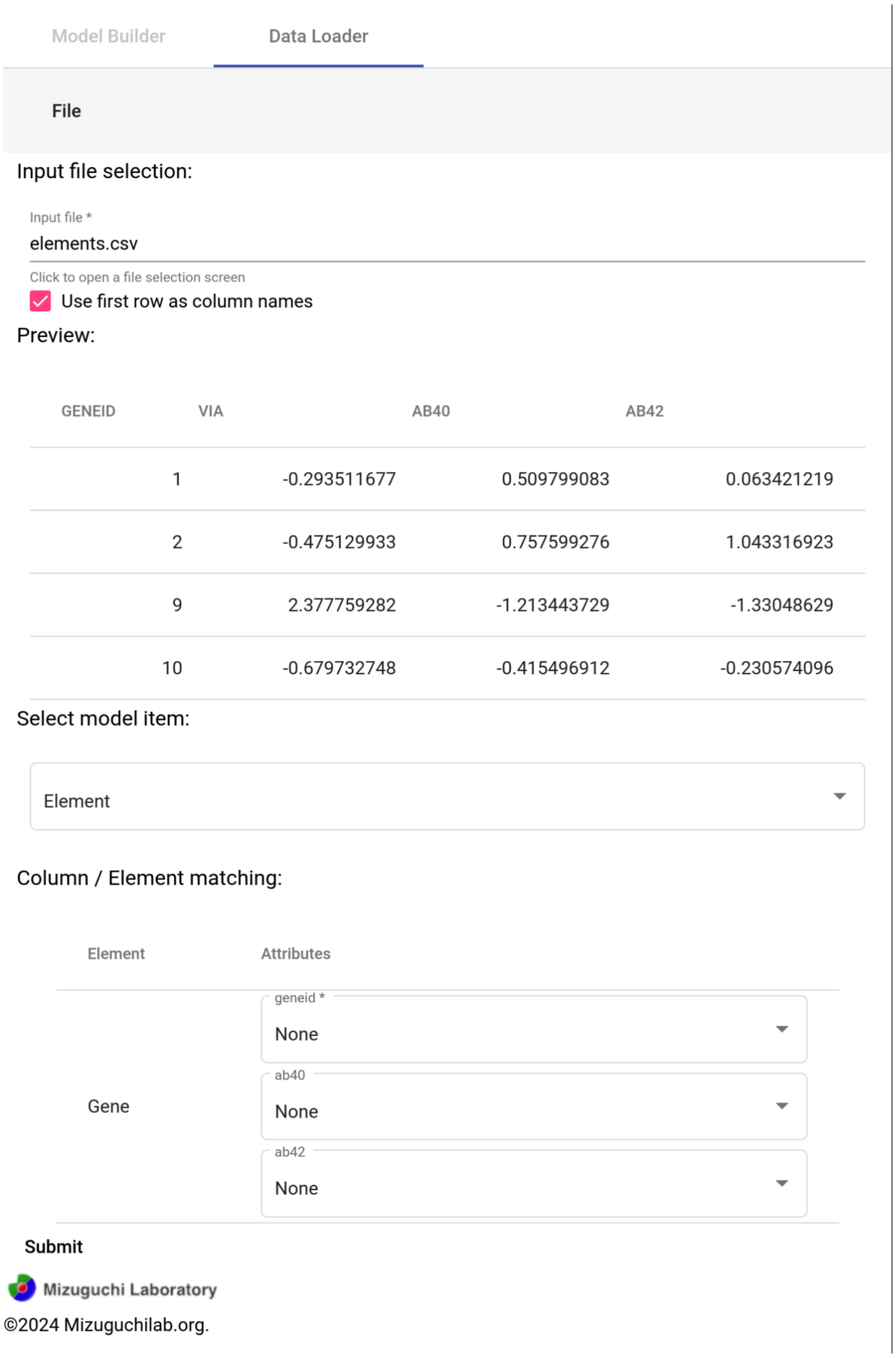

Figure 2-6. Different columns from the input file are matched to the attributes of an element for data upload.

Data associated with relations can be uploaded in the same fashion. Change the model items to `Relation` from the drop-down menu and follow the same steps as previously to upload relations data from files.

Data Persistance

For data to be automatically loaded by the *Data Enquirer* interface, we need to create a copy of the database used by the *Data Provider* interface. In order to generate the copy of the database, we use the `send_dump` command included in the definition of the `send_db` container as follows:

```
:~/sendProvider$ docker exec -it send_db send_dump
:~/sendProvider$ ls backup/
> backup.sql  elements.csv  model.json  relations.csv
```

Notice how running the command creates file `backup.sql`, that is stored under the current directory's `backup` subfolder, and that is a copy of the database currently being handled by the *Data Provider* interface.

Finish Execution

To terminate the execution of the *Data Provider* interface simply use the command `docker compose down` to bring down all the components in the required order:

```
:~/sendProvider$ docker compose down
[+] Running 4/4
 ✔ Container send_backend                Removed     10.5s
 ✔ Container send_provider               Removed      0.3s
 ✔ Container send_db                     Removed      0.3s
 ✔ Network send_dataprovider_send_net    Removed      0.2s
```

Model and Data Sharing

After exporting the model definition and obtaining a copy of the database with the populated model, these files need to be shared in order for potential users of the *Data Enquirer* interface to access the data.

Providing an exhaustive list of the available options for sharing the `model.json` and `backup.sql` files is beyond the scope of this user guide; however, and considering size limitations, these could be shared via email, uploaded to various sharing services such as Google drive or One drive, or made available through personal web pages.

Additional Sample Dataset

An additional, more complex data model is included in the GitHub distribution of the *Data Provider* interface. This model includes *Elements* representing Genes, Pathways and Go Annotations; and *Relations* in the form of genes and their corresponding annotations and protein-protein interaction networks, among others.

Both the model definition (`model.json` file) and the data needed to populate the model can be found under folder `other_examples/sample2_backup/` of the *Data Provider* repository.

3. Data Enquirer

Overview

The *Data Enquirer* interface is the element of SEND available for users who want to query data previously shared by other users. The interface is composed of three different components (each implemented as an independent software element); namely the `Database` component, the `Backend` component, and the `Data Enquirer` component. Notice that both the `Database` and `Backend` components are shared with the *Data Provider* interface.

A typical workflow for the *Data Enquirer* interface comprises the following steps:

1. Software installation (download)
2. Download and configuration of Data Model and Database backup.
3. Data download

In the following, we provide users with details on each of these steps, and provide sample models and database backup that can be used for functionality testing.

Also, in the same line with the *Provider interface,* we have also created a video companion for the steps we consider will mostly fit the potential users of SEND. The video for the *Enquirer interface* of SEND is available at: https://vimeo.com/1113272256

Installation

The *Data Enquirer* interface is available and ready-to-use through docker images[3]:

1. The download and execution of all the components that comprise the *Data Enquirer* interface can be done by using a single configuration file: a `docker-compose.yml` used for the component; and the files defining the model (`model.json`) and data (`backup.sql`) originally created and shared by users of the *Data Provider* interface.

[3] As with the Data Provider interface, an installation of docker is required to use this method. A guide to install docker is available from the official website at https://docs.docker.com/get-docker/

The configuration file is freely available at:
https://github.com/targetmine/send_DataEnquirer/blob/main/docker-compose.yml

Alternatively, the file can be downloaded using the following terminal commands:

```
:~/$ mkdir sendEnquirer
:~/$ cd sendEnquirer
:~/sendEnquirer$ wget
https://raw.githubusercontent.com/targetmine/send_DataEnquirer/refs/head
s/main/docker-compose.yml
```

2. Access the folder where the `docker-compose.yml` file was saved and create a sub-folder named `backup`. The model definition and the backup of the provided database need to be saved to this default directory for the *Data Enquirer* to load them properly.

```
:~/sendEnquirer$ mkdir backup
```

3. Download the `backup.sql` and `model.json`[4] files made available by a Data Provider and save them to the previously created `backup` directory. Alternatively, download the sample versions available at the `send_DataEnquirer/backup` repository either directly from GitHub's website or using the following commands:

```
:~/sendEnquirer$ cd backup
:~/sendEnquirer/backup$ wget
https://raw.githubusercontent.com/targetmine/send_DataEnquirer/refs/head
s/main/backup/model.json
:~/sendEnquirer/backup$ wget
https://raw.githubusercontent.com/targetmine/send_DataEnquirer/refs/head
s/main/backup/backup.sql
```

4. Execute the command: `docker compose up -d`.
The `docker-compose` file contains the instructions to (download and) execute the three containers that form the backbone of the *Data Enquirer* interface, namely the `Database`, `Backend` and `DataEnquirer` components. On execution, a docker volume for the internal handling of the system's database will be created. Also, the model and backed up database will be automatically loaded from the default location.

```
:~/sendEnquirer/backup$ cd ..
:~/sendEnquirer$ docker compose up -d
```

[4] Both the `backup.sql` and `model.json` files should be made available by the Data Provider user. Users can share these files through email, their own websites, or simple file sharing platforms (i.e. Google Drive). Files that can be used for testing the platform are available in the *Data Enquirer*'s GitHub repository.

```
[+] Running 5/5
 ✔ Network send_dataenquirer_send_net  Created           0.1s
 ✔ Volume "send_volume"                Created           0.0s
 ✔ Container send_db                   Started           0.5s
 ✔ Container send_backend              Started           0.8s
 ✔ Container send_enquirer             Started           0.7s
```

5. Once the three containers are started by the docker daemon, the application will be available through a normal web browser on address http://127.0.0.1:4220/

Source code for the *Data Enquirer* interface is publicly available on GitHub at https://github.com/targetmine/send_DataEnquirer. Notice that in order to run the interface from source, both the `send_DataEnquirer` and `send_Backend` repositories need to be downloaded.

The `Data Enquirer` component of SEND is implemented using the Angular framework. To compile and execute the source code, a working installation of *npm* and *Angular* are required.

In addition to *npm*, the `Backend` component of SEND also requires a working installation of PostgreSQL[5]. PostgreSQL needs to provide access to a database that matches the conditions set on the `.env` settings of the `Backend` component.

Compile and run the individual components in the required order, i.e. first the PostgreSQL database management component, then the `Backup` component, and finally the `Data Enquirer` component.

Model and Data downloading

The two different interfaces of SEND target two different types of users. In particular, the *Data Enquirer* interface is targeted at users that want to explore and download sections of the data *already made available by Data Providers.*

With this in mind, it is clear that it is indispensable for the *Data Enquirer* to have access to the model and data already shared by others. An extensive discussion on the means providers have to share the defined model and its data is beyond the scope of this guide; however, these include sharing the required files through email, the use of different hosting services or websites, or the use of traditional file sharing services such as Google Drive.

The GitHub repository for the *Data Enquirer* interface, under folder `backup`, makes available both a model and a database backup that can be used as a test when no other sources of data are available. The downloaded `model.json` and `backup.sql` files should be saved to a folder named `backup` located at the same level of `docker-compose.yml`.

[5] It is possible to provide the database through the use of an ad-hoc docker image.

Automatic model loading

As mentioned before, to avoid the need of users to code or perform database operations, the *Data Enquirer* interface of SEND loads the model (`model.json`) and data (`backup.sql`) stored in folder *backup*. A directory file structure like the one shown below is the minimum required to run the *Data Enquirer* interface:

```
:~/$ tree -a sendEnquirer/
sendEnquirer/
├── backup
│   ├── backup.sql
│   └── model.json
└── docker/composer.yaml

1 directory, 3 files
```

In particular, from folder `backup/` the interface loads the database model stored in the `model.json` file; and populates the database with the dump stored in file `backup.sql`. These two files should be, in principle, generated and shared by a user of the *Data Provider* interface of SEND. For testing purposes, there are versions of these two files available at the GitHub repository for the `Data Enquirer` component. After saving these files to the corresponding directory, and bringing the system up, the model and data displayed by the application should be like the one from Fig. 3-1.

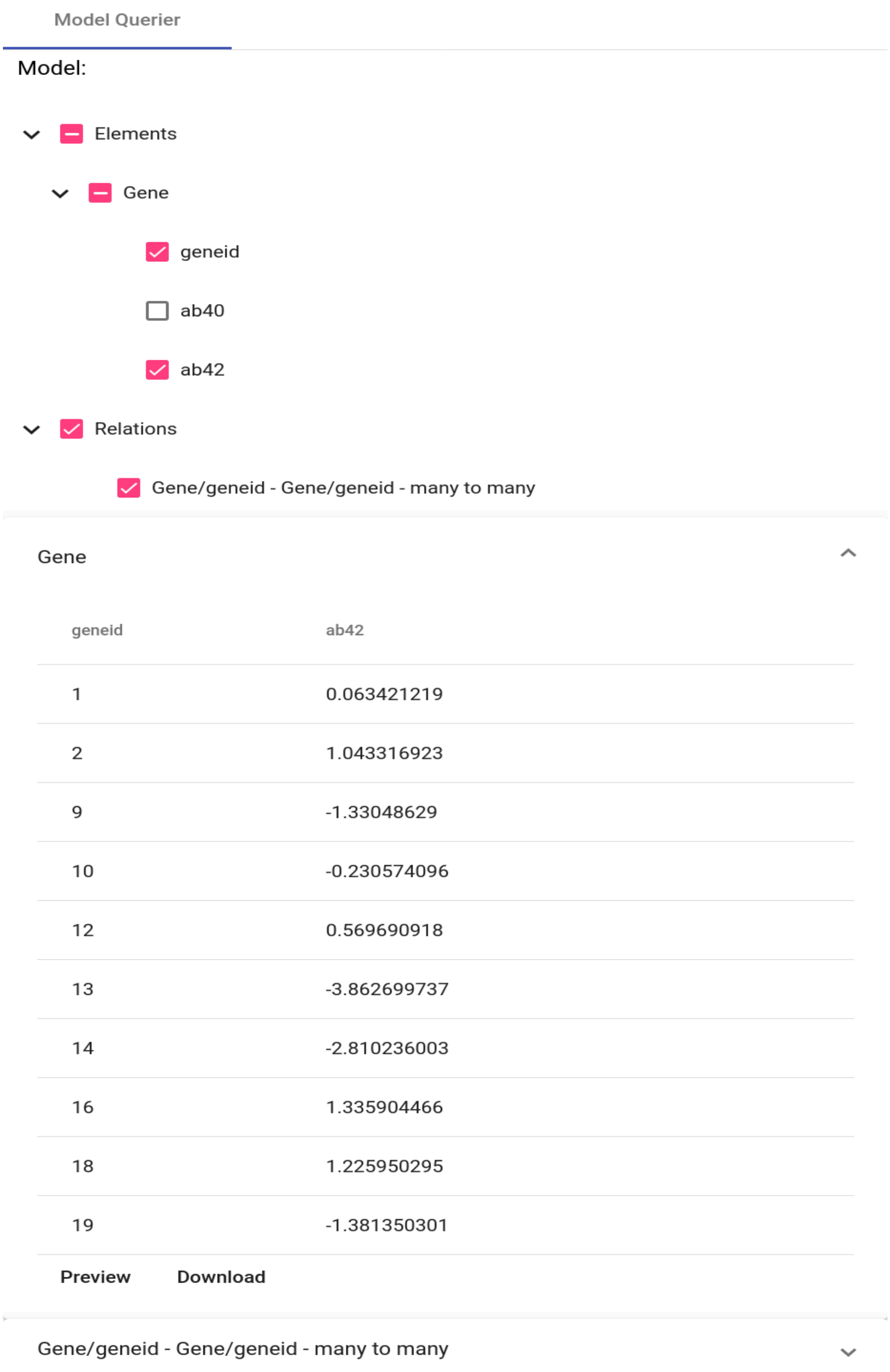

Figure 3-1. Data enquirer interface with the shared model.

Using the toggle display controls available, it is possible to navigate and inspect both the `Elements` and their attributes; together with the `Relations` included in the model.

Furthermore, after selecting different components from the model, it is possible to *preview* the first few instances of the selected fields stored in the underlying database. This can be achieved by simply clicking on the `Preview` button at the bottom of the drop-down that appears each time an `Element` or a `Relation` is selected. Notice that it is possible to preview both `Elements` and `Relations` simply one at a time.

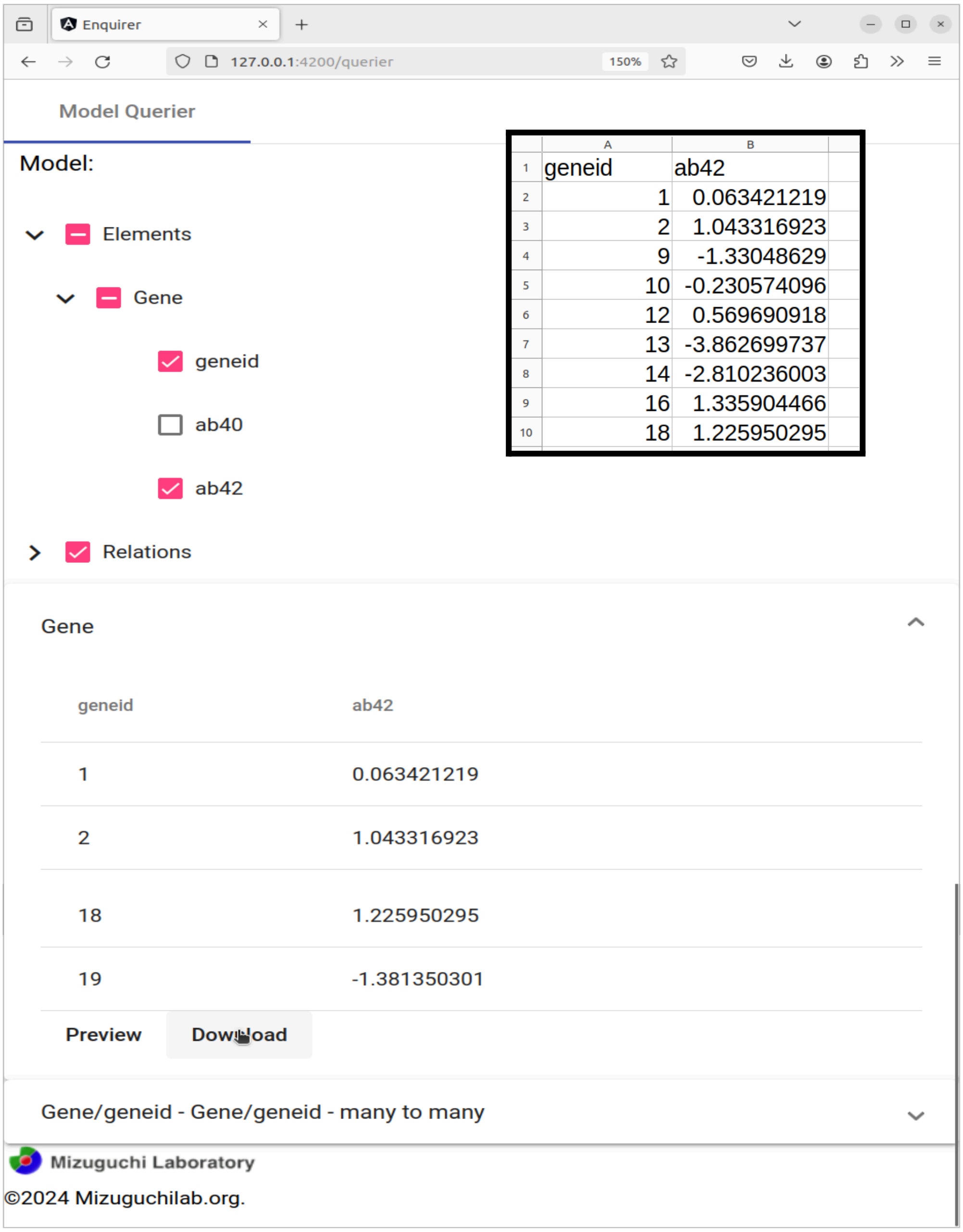

Figure 3-2. Select the Element and combination attributes required and click on `Download` *to retrieve a CSV version of the data available in the underlying database. Also shown a small section of the downloaded csv file.*

In a similar way, the different `Relations` available in the current model can also be downloaded to local copies by simply selecting them and clicking on the corresponding `Download` button, as shown in Fig. 3-3.

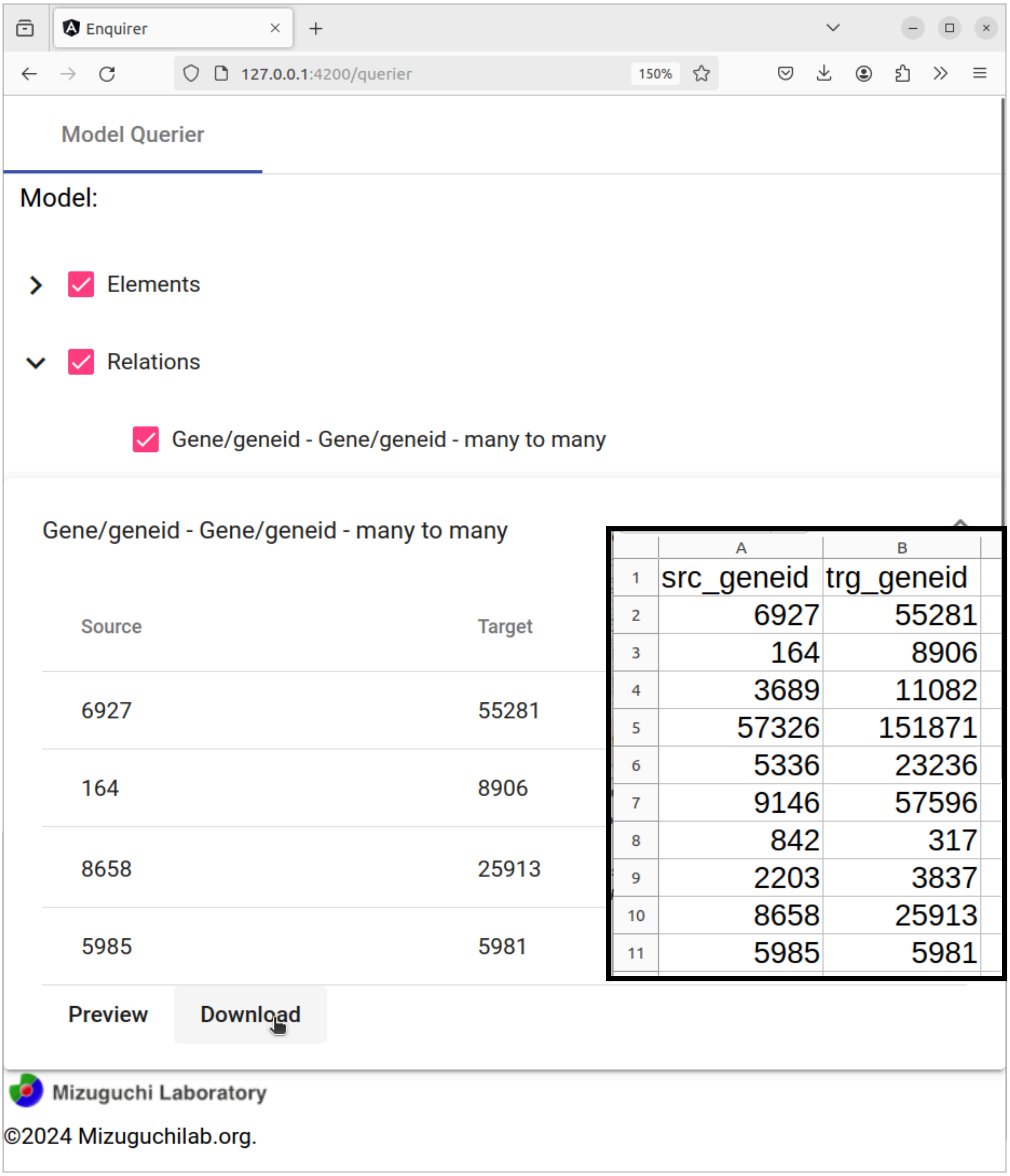

Figure 3-3. Relations can also be selected and downloaded using the `Download` available at the bottom of the preview display. Also shown a small section of the downloaded csv file.

Notice that data will be downloaded using the browser's default saving dialog, thus different browsers will potentially behave differently depending on the current settings. By default, the data selected for download will be saved to a file named `data.csv`, located at the browser's default download destination folder.

Finish Execution

To finish the execution of the *Data Enquirer* interface, simply use the command `docker compose down` to bring down all the components.

```
:~/sendEnquirer$ docker compose down
[+] Running 4/4
 ✔ Container send_backend                Removed     10.9s
 ✔ Container send_enquirer               Removed      0.7s
 ✔ Container send_db                     Removed      0.8s
 ✔ Network send_dataenquirer_send_net    Removed      0.5s
```

Additional Sample Dataset

An additional test dataset is included with the *Data Enquirer* interface as part of the GitHub distribution, to serve as a complement to the one included in the *Data Provider* interface. Notice that, although the model continues to include the same *Elements* and *Relations* previously described, the *Enquirer* distribution only includes the model definition (`model.json` file) and a copy of part of the uploaded data (`backup.sql` file).

Both files can be found under folder `other_examples/sample2_backup/` of the *Data Enquirer* repository.

4. Backend

Overview

The `Backend` component of SEND is responsible for providing the connection between the PostgreSQL database where models and data are stored, and both the *Data Provider* and *Data Enquirer* interfaces.

Installation

The recommended way to use the `Backend` component of Send is through ready-to-use docker images. The `Backend` component will normally be downloaded and executed automatically when using the `docker-compose.yml` file available for both the *Data Provider* and *Data Enquirer* interfaces. No additional installation is needed.

Using source code (Optional - Advanced Users)

Advanced users can run the `Backend` component of SEND directly from source code. Notice that a PostgreSQL instance needs to be executed prior to running this component. The recommended way to use PostgreSQL is through the Docker images made available directly by the publishers.

1. Use the following commands to start the network and volume elements of Docker required by the PostgreSQLdoc database and the `Backend` component:

```
$ docker network create -d bridge send_net
$ docker volume create send_volume
```

2. Use the following commands to download the latest PostgreSQL image available and start it with the configuration required by the `Backend` component:

```
$ docker run -it --rm -d -p 5433:5432 \
> -e POSTGRES_PASSWORD=example \
> -e PGDATA=/var/lib/postgres/data \
> --mount type=volume,src=send_volume,target=/var/lib/postgres/data \
> --network send_net \
> --name datasharing_db postgres
```

3. Use the following commands to clone the source code for the `Backend` component from GitHub and enter the corresponding directory:

```
$ git clone https://github.com/targetmine/send_Backend.git
$ cd send_Backend
```

4. Finally, use the following sequence of commands to run the `Backend` component:

```
$ npm install
$ npm run build
$ npm start
app is listening on port 5000!
```

When correctly executed, a web browser should display the message `API is alive` when accessing localhost on port 5000.
